# PRDM9, a driver of the genetic map

**DOI:** 10.1371/journal.pgen.1007479

**Published:** 2018-08-30

**Authors:** Corinne Grey, Frédéric Baudat, Bernard de Massy

**Affiliations:** Institut de Génétique Humaine, Centre National de la Recherche Scientifique, University of Montpellier, Montpellier, France; Cornell University, UNITED STATES

## Abstract

During meiosis, maternal and paternal chromosomes undergo exchanges by homologous recombination. This is essential for fertility and contributes to genome evolution. In many eukaryotes, sites of meiotic recombination, also called hotspots, are regions of accessible chromatin, but in many vertebrates, their location follows a distinct pattern and is specified by PR domain-containing protein 9 (PRDM9). The specification of meiotic recombination hotspots is achieved by the different activities of PRDM9: DNA binding, histone methyltransferase, and interaction with other proteins. Remarkably, PRDM9 activity leads to the erosion of its own binding sites and the rapid evolution of its DNA-binding domain. PRDM9 may also contribute to reproductive isolation, as it is involved in hybrid sterility potentially due to a reduction of its activity in specific heterozygous contexts.

## Introduction

Sexual reproduction requires the conversion of diploid cells into haploid cells through a specialized cell cycle that is called meiosis. Homologous recombination events occurring during meiosis generate new combinations of alleles; therefore, the genetic material transmitted to the next generation is distinct from the one of the parental chromosomes. By increasing genetic diversity, homologous recombination has long-term consequences on genome evolution. Moreover, it is also an obligatory step for successful meiosis. Upon completion of reciprocal homologous exchanges, homologous chromosomes (homologues) are physically connected, allowing their proper orientation for segregation at the first meiotic division. Defects or absence of meiotic recombination can lead to mis-segregation, aneuploidy, and sterility [1].

**Fig 1:**
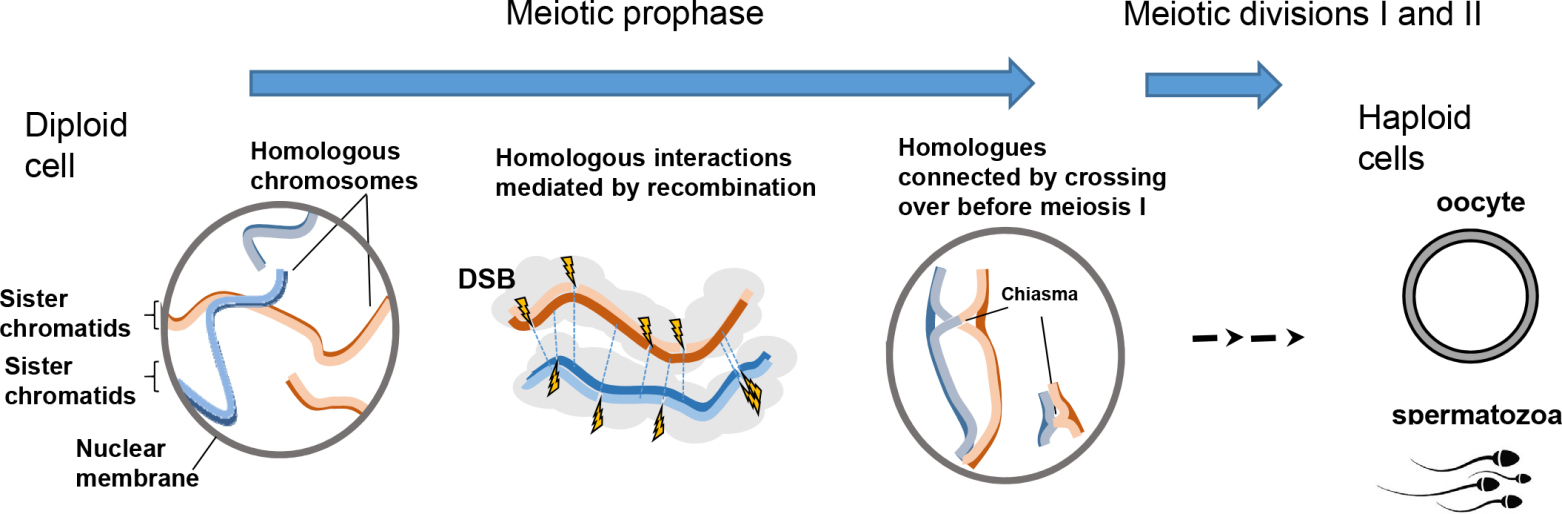
The role of homologous recombination during meiotic prophase. During meiotic prophase, homologous recombination allows the interaction, alignment, and connection through chiasmata of homologous chromosomes. Chromosomes are structured by a protein axis (blue and red lines for paternal and maternal chromosomes, respectively) to which chromatin loops (grey cloud) are anchored. The recombination pathway is initiated by the formation of several DSBs (yellow lightning) on each homologue. DSB sites are determined by PRDM9. DSB repair promotes interactions between homologues (thin blue dotted lines). A small subset of DSB repair events, at least one per homologue pair, leads to a crossover visualized as a chiasma that establishes a topological connection between homologues. These connections are required for proper chromosome segregation at the first meiotic division (meiosis I). The two meiotic divisions lead to the formation of haploid oocytes and spermatozoa. DSB, DNA double strand break; PRDM9, PR domain-containing protein 9.

At the molecular level, meiotic recombination is initiated by the induction of programmed DNA double strand breaks (DSBs) that are repaired by homologous recombination, leading to gene conversion and crossing over. The meiotic homologous recombination pathway requires a homology search and the formation and resolution of joint molecules preferentially formed between homologues rather than sister chromatids [2]. DSB localization and the pathway choices during DSB repair are thus important factors that determine where exchanges between genetic loci take place (Fig 1). In mice, primates, and other vertebrates, DSB localization occurs at genomic sites that are bound by the sequence-specific DNA-binding domain of PR domain-containing protein 9 (PRDM9) [3–6]. The control of the recombination landscape by PRDM9 has many implications in terms of the molecular mechanisms underlying the interaction between homologues during meiotic prophase and genetic diversity. Here, we report the current knowledge about the molecular features of PRDM9 as a DNA-binding protein and epigenetic modifier and how those features contribute to drive DSB formation to PRDM9-binding sites. We also discuss the consequences of PRDM9 activity on reproductive isolation and hybrid sterility.

## PRDM9 structure and function

**Fig 2:**
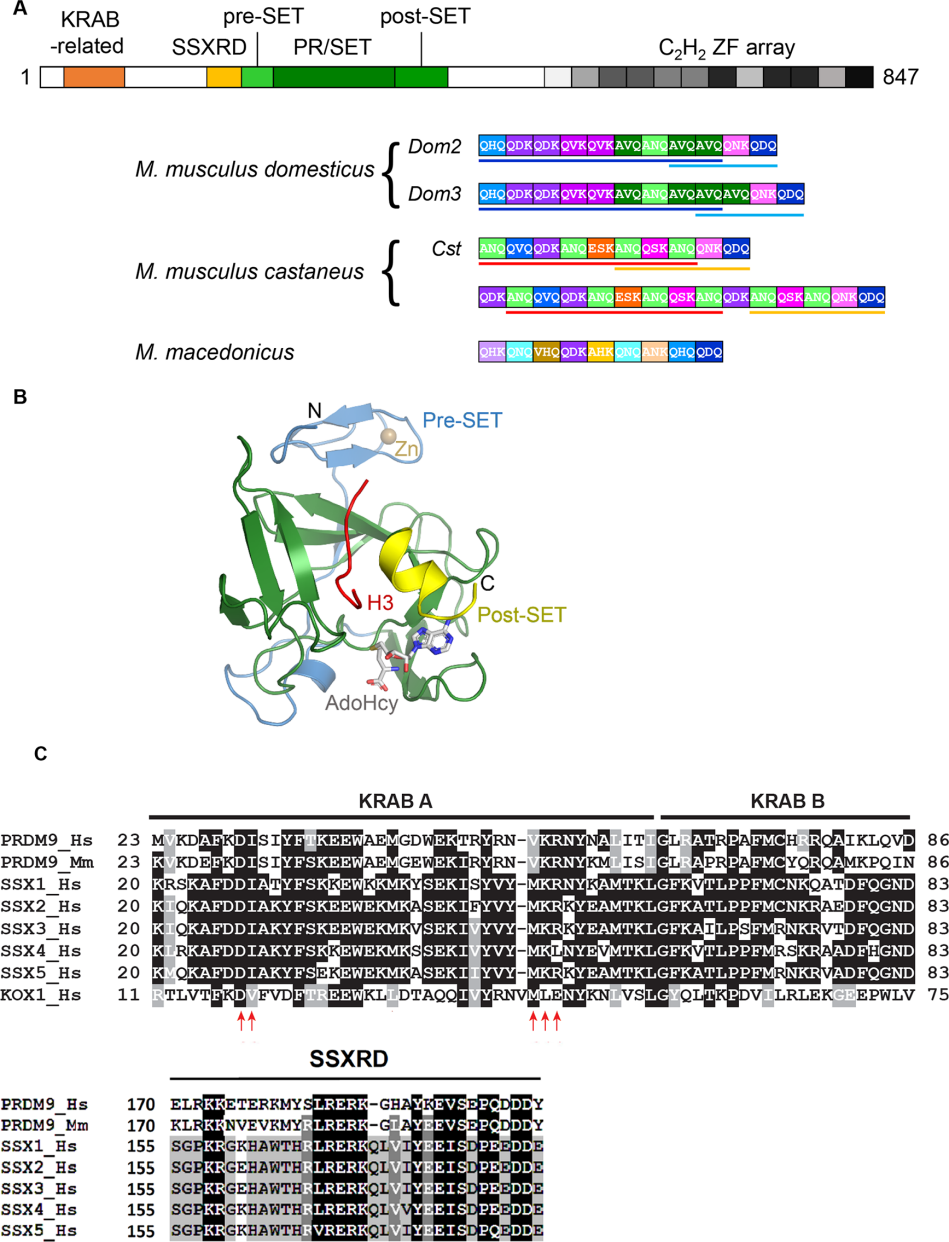
PRDM9 domains. (A) The domain organization of PRDM9 and the high diversity of its zinc finger array. Top, schematic representation of the mouse PRDM9 protein. Bottom, zinc finger arrays from two *Mus musculus domesticus* alleles (*Dom2* and *Dom3* from C57BL/6 and C3H lab strains, respectively), two *M*. *m*. *castaneus* alleles (including *Cst* from CAST strain) and one representative allele from *Mus macedonicus* (all alleles described in [31]) are shown underneath. Each box represents one zinc finger, and each color represents a specific zinc finger sequence. The DNA-contacting amino acids in position −1, 3, and 6 of the zinc finger are indicated within each box. Colored bars underline blocks of zinc fingers conserved between alleles from the same subspecies. The recurrence of several individual zinc fingers is apparent within each array. Alleles from the same species (*M*. *musculus*) are largely made of combinations of the same set of zinc fingers; conversely, a majority of zinc fingers is not shared between *M*. *musculus* and *M*. *macedonicus*. (B) Structure of the mouse PRDM9 PR/SET domain (residues 198–368) in complex with a H3K4me2 peptide (H3) and AdoHcy. The SET domain (residues 245–358) is shown in green, the pre-SET domain in blue, and the truncated post-SET in yellow (gift from Jan Kadlec). Pre-SET and post-SET are a zinc knuckle and a zinc finger, respectively, involved in the organization of the SET domain. (C) The KRAB-related and SSXRD domains. PRDM9 shares similarity with the KRAB-related and SSRD domains of the SSX protein family (SSX1 to 5). KOX1 that contains a canonical KRAB domain and interacts with TRIM28 is also shown. Amino acid substitutions that abolish the interaction between KOX1 and TRIM28 are shown by red arrows (reprinted from [49]). The alignment shows similarities in these two domains (white on black: 100% identity, white on grey: 80% identity, black on grey: 60% identity, black on white: less than 60% identity). AdoHcy, Adenosyl-homocysteine; CAST, CAST/Eij mouse strain; H3K4me2, Histone H3 lysine4 dimethyl; Hm, *Homo* sapiens; KOX1, Krüppel-associated box 1 protein; KRAB, Krüppel-associated box; Mm, *Mus musculus*; PRDM9, PR domain-containing protein 9; SET, Suppressor of variegation 3–9, Enhancer of Zeste and Trithorax; SSXRD, synovial sarcoma, X breakpoint repression domain; TRIM28 Tripartite motif-containing 28.

PRDM9, which is only expressed in oocytes and spermatocytes [7], is a member of the PRDM family of transcription factors [8–10]. PRDM9 is composed of three main regions that include conserved domains with specific molecular activities (Fig 2).

- The amino-terminal region comprises two domains thought to mediate protein interactions: a Krüppel-associated box (KRAB)–related domain and a synovial sarcoma, X breakpoint repression domain (SSXRD) [11].
- The central region contains the PR/SET domain named after positive regulatory domain I-binding factor 1, also called PRDM1 (PRDI-BF1) and retinoblastoma protein-interacting zinc finger gene 1, also called PRDM2 (RIZ1) homology. This domain is distantly related to the family of Suppressor of variegation 3–9, Enhancer of Zeste and Trithorax (SET) domains found in many histone methyltransferases [12].
- The C-terminal region is made of a DNA-binding domain, which is composed of an array of Cysteine(2), Histidine(2) (C2H2) zinc fingers in tandem and is encoded by a single exon [13]. The association of the PR/SET domain with the C2H2 zinc fingers characterizes the PRDM family [8–10].

### The DNA-binding domain of PRDM9

C2H2-type zinc fingers are a structure that can bind to DNA in a sequence-specific manner and are found in the large transcription factor family of zinc finger proteins [14, 15]. The PRDM9 tandem array of zinc fingers displays the archetypical architecture of tandem C2H2 zinc finger domains [16] in which successive zinc fingers are separated by a conserved linker to allow binding of each zinc finger to adjacent double strand DNA triplets [17, 18].

Several algorithms have been used to predict the DNA-binding sites of PRDM9 from the primary sequence of the zinc fingers, based on the fact that the specificity of C2H2 zinc finger interaction with DNA is mediated essentially by the identity of three or four residues at specific positions in the zinc finger [19–21]. These methods have led to the identification of DNA sequence motifs that resemble the sequences actually bound by PRDM9. For instance, it was correctly predicted that the most common human PRDM9 variant (PRDM9^A^) binds to a 13-mer motif that is enriched at human hotspots [3, 4, 22]. Nevertheless, in silico predictions do not have the power to reliably identify genomic sites bound in vivo by zinc finger proteins, including PRDM9 [15]. This inability could partly result from the complexity of these composite domains in which it is difficult to know the relative role of every zinc finger and the interactions between them. In addition, factors other than the DNA sequence could also be involved (see paragraph below).

In vitro testing of PRDM9 binding to sequences localized at the center of mouse and human recombination hotspots using various techniques (southwestern blotting [3], electrophoretic mobility shift assay [3, 23], Affinity-seq [24], fluorescence anisotropy [16], switchSENSE [25]) led to two important conclusions. First, PRDM9 binds specifically to sequences localized at the center of recombination hotspots. Second, polymorphisms in the PRDM9-binding sites that alter the affinity for PRDM9 have parallel effects on PRDM9 binding and recombination frequency. However, the effects of specific polymorphisms on PRDM9 affinity remain difficult to predict, as exemplified by the finding that the same individual zinc finger displays different sequence specificity depending on its position along the array [23].

Fragments of two human PRDM9 zinc finger arrays have been crystalized in complex with their double-stranded DNA target sequences [16, 26]. The structure of this complex sheds light on several DNA–amino acid interactions and their specificity; for example, it revealed that several invariant Cytosine:Guanine (C:G) base pairs are recognized primarily by conserved histidine or arginine residues of the zinc finger α-helix. Conversely, PRDM9–DNA interaction may be stabilized by hydrogen bonds with variable base pairs, giving PRDM9 some adaptability to sequence variations. Specific PRDM9 zinc finger variations found to alter the in vivo crossover frequency at two human hotspots did not always correlate with in vitro measured affinity variations, although these last measurements were essentially in line with predictions based on the structure. This could result either from in vitro assays being made with only fragments of the zinc finger array or from a regulation of site accessibility in spermatocyte nuclei [16, 27].

Additional factors also play a role in PRDM9 binding, such as the local chromatin environment, which is determined by the histone content of nucleosomes, their post-translational modifications, and their positioning. These chromatin properties could modulate the accessibility of DNA sequences to PRDM9. Indeed, PRDM9-binding sites in mice have been determined in vivo by chromatin immuno-precipitation followed by sequencing (ChIP-Seq) and compared to in vitro binding analyzed by Affinity-seq [24], showing that its binding is negatively influenced by heterochromatin histone marks. Strikingly, only about half of the sites identified in vitro were detected in vivo, and domains enriched for H3K9me2 (histone H3 lysine9 dimethyl), H3K9me3, or Lamin B1 were depleted for PRDM9-binding sites in vivo [24]. The influence of chromatin on PRDM9 binding may partly explain the differences in crossover [28, 29] and DSB activity [30] between oocytes and spermatocytes that show distinct nuclear, chromosomal, and chromatin organizations.

A striking property of the PRDM9 zinc finger array is its rapid evolution (discussed in section 3). Consequently, the PRDM9 zinc finger array not only displays a high level of diversity within species but is also not shared between species (Fig 2A). Large numbers of alleles with variable zinc finger content (3 to 19) and identity, with various predicted DNA-binding specificities, have been identified in several species, such as mice (>80 different alleles [31–33]), equids [34], bovids [35, 36], several species of nonhuman primates [37–40], and humans [3, 6, 27, 28, 41, 42].

### PRDM9 methyltransferase domain

The PR/SET domain of PRDM9 is distantly related to the family of the SET domains found in many histone methyltransferases [8, 9]. It was first reported that PRDM9 can catalyse trimethylation of H3K4me2 [7]. It was subsequently shown that isolated mouse and human PRDM9 PR/SET domains and full-length mouse PRDM9 can catalyse mono-, di-, and trimethylation of H3K4 and H3K36 in vitro [43–46]. The mouse PRDM9 PR/SET domain possesses also intramolecular automethylation activity, which could participate in regulating PRDM9 histone methyltransferase activity by modulating PR/SET domain folding [47]. Despite its relatively poor sequence conservation, the active form of this domain (in complex with a H3K4me2 peptide and adenosyl-homocysteine) adopts a structure similar to that of canonical SET domains [46]. This structure allowed identifying several key residues for PRDM9 catalytic activity, including the conserved tyrosine 357 (Y357) that is absolutely required for in vitro methyltransferase activity (Fig 2B) [46]. The PR/SET domain of PRDM7, which is the result of a duplication of the *PRDM9* gene specific to primates [13], contains the Y357S mutation and shows only residual H3K4 methyltransferase activity [48].

### PRDM9 KRAB-related and SSXRD domains

In its N-terminal region, PRDM9 contains two adjacent domains, a KRAB-related and a SSXRD domain (Fig 2C). These two domains are also present in SSX proteins. The KRAB-related domain is the ancestor of the KRAB domain found in KRAB zinc finger proteins but with distinct properties [11]. In particular, the KRAB-related domains of PRDM9 and of SXX1, a member of the SSX protein family, appear not to interact with the KRAB binding protein tripartite motif-containing 28 (TRIM28) [16, 49, 50]. This lack of interaction may be due to the absence of motifs in the KRAB-related domain, which are conserved and shown to be important for interaction with TRIM28 in (canonical) KRAB domain proteins [51]. TRIM28 in association with the KRAB domain of KRAB zinc finger proteins is forming a transcription repressor complex [52]. In contrast, the KRAB-related domain of SSX1 and SSX2, two members of the SSX family, have only weak transcriptional repression activity [50]. Although the SSXRD domain of SSX1 and 2 alone display transcription repression activity [50], the region including the KRAB-related and SSXRD domains of human PRDM9 does not show any repressive activity [53]. Thus, PRDM9 may not have any specific role in transcriptional regulation despite some changes at the RNA level that were detected in *Prdm9*^−*/*−^ mice [54] but may result from indirect consequences of the meiotic prophase arrest. Nevertheless, the KRAB-related domain of PRDM9 is involved in protein interactions as shown recently by identification of several partners interacting with this domain [49, 55]. N-terminal truncation of mouse PRDM9, including part of the KRAB-related domain, leads to a loss of function phenotype [49].

## From PRDM9 binding to DSB formation

### PRDM9 determines the localization of meiotic recombination sites

Several lines of evidence have led to the conclusion that, in meiotic cells, PRDM9 binds to specific DNA motifs in the genome and promotes histone modifications (H3K4me3 and H3K36me3) through the methyltransferase activity carried by its PR/SET domain. This leads to the recruitment of proteins required for the formation of DSBs in the vicinity of its binding site. PRDM9 is thus a specifier of sites of meiotic recombination.

**Fig 3:**
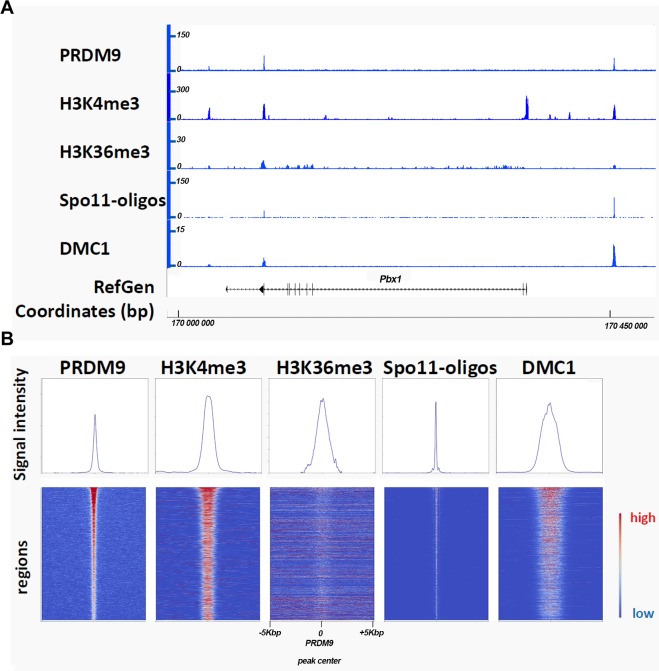
Sites of meiotic DSB formation. (A) Detection of PRDM9, H3K4me3, H3K36me3, SPO11-oligos, and DMC1 in spermatocytes from C57BL/6 mice. A 500-kbp chromosomal region on mouse Chromosome 1 shows typical PRDM9-dependent DSB sites (1–2 kb wide) and enrichment for reads obtained after PRDM9 [59], H3K4me3 [77], H3K36me3 [59], and DMC1 [59] ChIP-Seq and after SPO11-oligo purification [64]. These sites also contain DNA sequences that share similarity with the PRDM9 consensus motif (PRDM9^Dom2^ in this case) (not shown). An annotated gene (*Pbx1*) and coordinates (bp) are shown in the lower part. (B) Average plots (upper panels) and heatmaps (lower panels) of PRDM9, H3K4me3, H3K36me3, SPO11-oligos, and DMC1 ChIP-Seq. 2,601 regions of PRDM9^Dom2^ (the PRDM9 allele expressed in C57BL/6) binding in the C57BL/6 strain [59] were pooled (average plots) or ranked relative to the strength of the PRDM9 signal. In the heatmaps, each line indicates a genomic region of 10 kbp centered on the peak of PRDM9 binding where the signal intensity along that region (reads recovered by NGS) is represented by a color code (red, highest signal; blue, lowest signal). H3K36me3 is blurred due to overlapping signals from transcription activity. The plots show the average values over the 10-kbp interval for all the 2,601 regions. DSB, DNA double strand break; ChIP-Seq, chromatin immuno-precipitation followed by sequencing; H3K4me3, histone H3 lysine4 trimethyl; NGS, next generation sequencing; PRMD9, PR domain-containing protein 9; SPO11, sporulation protein 11.

The analysis of specific recombination sites in the mouse genome [56–58] and population studies in humans [22] led to the proposition of this mechanism and the discovery of the role of PRDM9 [3, 4, 6]. Further work allowed genome-wide mapping of various molecular events that characterize meiotic DSB hotspots in mice, notably the detection of PRDM9 binding in mouse spermatocytes [24, 59, 60], the histone modifications H3K4me3 and H3K36me3 associated to PRDM9 activity [45, 59, 61–63], the formation of DNA breaks by SPO11 [64], and the presence of the DSB repair proteins DMC1 and RAD51 [62, 63] (Fig 3A and 3B). In humans, similar evidence has been obtained by the detection of H3K4me3 and DMC1 at specific genomic sites [65]. The development of a technique for mapping and quantifying DSB formation by DMC1 ChIP was instrumental for some of these analyses [63, 66]. In addition, consensus DNA motifs sharing similarity with the in vitro predicted PRDM9 zinc finger-recognition sequences have been identified at these initiation sites of meiotic recombination [62, 65].

Most, if not all, meiotic recombination events are controlled by PRDM9. This has been specifically shown in mice where changes in the PRDM9 zinc finger domain lead to the complete redistribution of hotspots [62]. It was suggested that one region in the mouse genome (the Pseudo Autosomal Region [PAR]) has PRDM9-independent hotspot activity. However, the PAR contains many repeated sequences, and this observation remains to be confirmed using a fully assembled sequence. In humans, hotspots in the PAR are determined by PRDM9 [65, 67, 68].

The initial finding of PRDM9-mediated DSB localization in mice and humans has been extended to other primates [39, 40, 69]. PRDM9 is also predicted to specify meiotic recombination sites in other vertebrate species [70], such as equids [34], bovines [35, 36], some fish species, turtles, snakes and lizards, and coelacanth [71]. The specific role of PRDM9 in defining the sites of meiotic recombination but not in DSB formation, per se, was demonstrated by the *Prdm9*^−*/*−^ mouse phenotype. In these mutant mice, which are sterile, meiotic DSBs are formed but at alternative locations, as described in Box 1.

**Box 1. DSB formation in the absence of PRDM9**

PR domain-containing protein 9 (PRDM9) is present only in some vertebrates and has been lost in others [71]. It is also absent in other organisms, such as fungi and plants. This led to the conclusion that there are two main pathways for the localization of meiotic recombination. In the absence of PRDM9, chromatin accessibility, potentially associated with other chromatin features, is a major determinant of DNA double strand break (DSB) sites. This has been studied in great detail in the yeast *Saccharomyces cerevisiae* in which DSBs preferentially take place in nucleosome-depleted regions adjacent to transcription promoters and to histone H3 lysine4 trimethyl (H3K4me3)-enriched nucleosomes [128]. In yeast, H3K4me3 has a direct molecular function in DSB formation because it interacts with Spp1, a member of the complex of proteins associated with SET1 (COMPASS) that also interacts with Mer2, an essential component for DSB formation [98, 99]. In dogs, some fish, and birds, for instance, in which *Prdm9* is nonfunctional or absent, meiotic hotspots preferentially localize to functional genomic elements enriched in H3K4me3, such as transcription start sites and/or unmethylated CytosineGuanine dinucleotide (CpG) islands [71, 129–134]. Differently from PRDM9-directed sites, conserved DNA motifs are not required for this localization, and the impact of the DNA sequence on hotspot activity, if any, could extend to multiple positions within and outside of the region covered by gene conversion tracts. Thus, biased gene conversion associated with DSB repair should not or only weakly influence hotspot activity. Strikingly, in organisms lacking *Prdm9*, the position of meiotic hotspots is conserved between related species, and this conservation can extend over tens of millions of years of evolution, indicating the stability of recombination sites [130, 133, 135]. Interestingly, when *Prdm9* is inactivated in mice, DSBs still occur but at new locations called “default sites” that mainly correspond to promoter regions and are also enriched in H3K4me3, thus resembling the situation in organisms without PRDM9 [62]. It has been proposed that CXXC finger protein 1 (CXXC1), the mouse orthologue of Spp1, is involved in mediating interactions between H3K4me3 (deposited by PRDM9 or not) and the DSB machinery [49]. Despite efficient DSB formation in *Prdm9*^−*/*−^ mice, these mice are sterile and show synapsis defects [7, 76]. The nature of the molecular defect is not known and could be a defect of DSB repair and/or misregulation of DSB formation (timing, efficiency).

### In vivo PRDM9 binding

**Fig 4:**
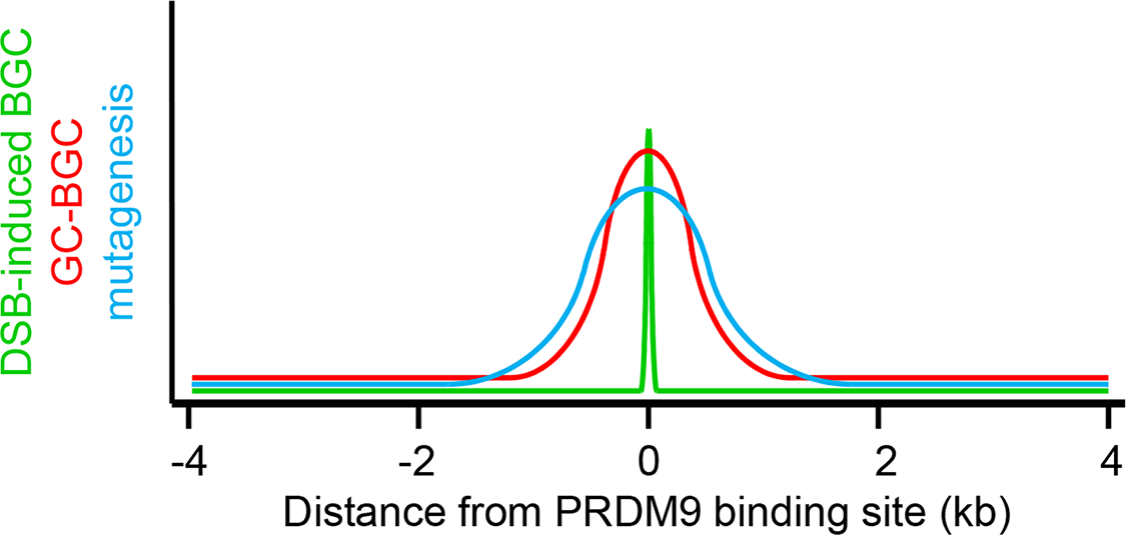
Long-term consequences of recombination activity. In addition to allele reshuffling between distant loci generated by crossovers, recurrent recombination events in PRDM9-defined hotspots influence locally the evolution of genome sequences within populations. The first consequence is the result of the molecular mechanism of recombination that leads to gene conversion of the region surrounding the initiating DSB in the allele of the noninitiating chromosome [104, 136] (Fig 7). Therefore, when a polymorphism that alters the initiation rate is within the frequently converted interval of a recombination hotspot, the allele associated with a higher initiation rate is undertransmitted. This phenomenon, called dBGC, might act against the emergence of new hotspots in the population and also favor the fixation of the less active alleles at existing hotspots, eventually leading to their extinction. It has been demonstrated that dBGC drives the erosion of PRDM9-binding motifs at hotspots (discussed in section 3.1) and might influence the base composition at the center of hotspots depending on the PRDM9-binding motif sequence. gBGC is the consequence of a bias of the recombinational repair of meiotic DSBs that favors the transmission of GC over AT alleles [137]. gBGC results in a rise in the frequency of GC alleles at polymorphic sites in populations and promotes their fixation. This bias in favor of the fixation of GC alleles is a signature of recombination hotspots, detectable as a local increase in the equilibrium GC–content. gBGC has been described in several species in which PRDM9 specifies recombination hotspots [59, 65, 107, 138, 139]. It has been proposed that the mechanism of recombination, because it involves some DNA synthesis, could increase locally the mutation rate [140, 141]. Support for increased mutagenesis at recombination hotspots comes from the higher diversity [142], from base compositions skews observed at DSB hotspots in mice and humans [65, 72, 138], and from direct measurement at one human hotspot [143]. dBGC, DSB-induced biased gene conversion; DSB, DNA double strand break; gBGC, GC-biased gene conversion; GC, gene conversion; PRDM9, PR domain-containing protein 9.

**Fig 5:**
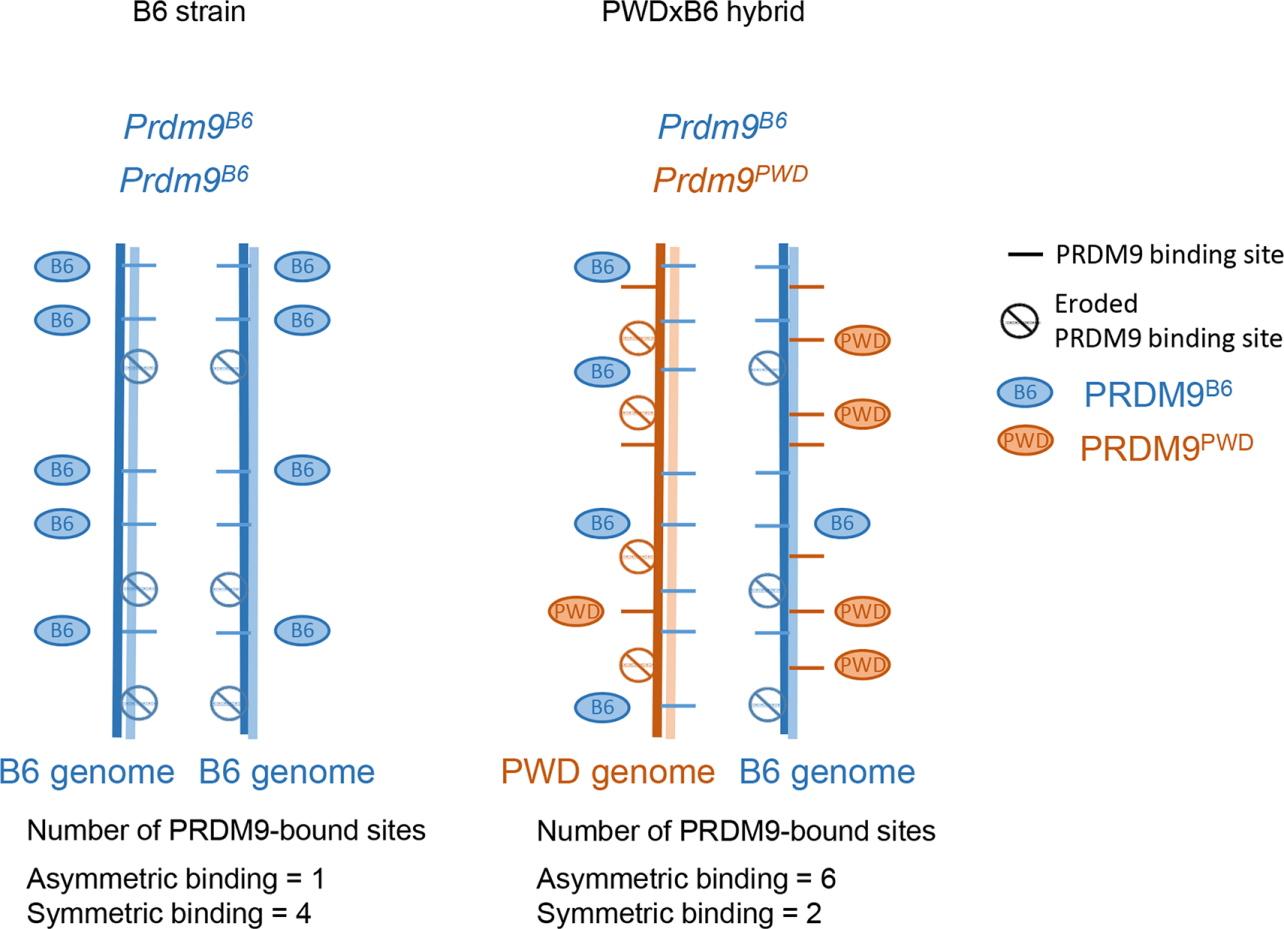
Heterozygosity at *Prdm9* and genetic incompatibility. When *Prdm9* is heterozygous (two alleles with different zinc finger domains), DSB sites can be specified by either allele, and the contribution of each allele to DSB formation is not always even but can vary from 50/50 due to the expression level of *Prdm9* alleles and/or to the density, affinity, or accessibility of binding sites. Due to hotspot erosion and to a lesser extent to mutations (see Fig 4), in hybrids between strains with divergent genomes, such as *Mus musculus domesticus* and *M*. *m*. *musculus*, and that have been in contact with specific *Prdm9* alleles for generations, some PRDM9 binding sites have been eroded specifically on one genome and are thus heterozygous. An example of a hybrid between the PWD and C57BL/6 (B6) strains (PWDxB6) is shown (right panel). It has been hypothesized that PRDM9 plays a role in DSB repair and that PRDM9 binding on both homologues is required for efficient DSB repair and proper homologous synapsis [73]. According to this assumption, heterozygous sites will differ from homozygous ones by having only one homologue bound with PRDM9. However, if PRDM9 level and/or affinity is low, PRDM9 may bind only on one homologue even at homozygous sites (B6, left panel). Such *Prdm9* heterozygous contexts have been described in both humans and mice, but they usually do not seem to influence meiosis and fertility [60, 62, 65, 72, 73, 75]. DSB quantification by DMC1 ChIP-Seq [73] revealed that heterozygous sites (also called asymmetric) have higher levels of DMC1, and this was interpreted as a delay or lower efficiency in DSB repair. The alternative possibility is that more DSBs are induced at such heterozygous sites. In one specific intersubspecific hybrid mouse with a specific genetic background (the PWDxB6 hybrid in which PWD represents *M*. *m*. *musculus* and B6 *M*. *m*. *domesticus*) and in which two different *Prdm9* alleles are present, males are sterile. This hybrid sterility context was discovered [144] and analysed in details by J. Forejt’s group [78, 145], who proposed that *Prdm9* could be a speciation gene [146]. The proportion of DSB events within asymmetric sites in PWDxB6 hybrids reaches 72%. This is higher than in other hybrids and suggests that a threshold of DSBs made at sites of symmetric PRDM9 binding is not reached in this context, resulting in the synaptic defect and sterility [73, 117]. It was also observed that the proportion of DSBs in “default sites” is increased in these hybrids compared to parental strains [72]. This could provide an alternative interpretation for the DSB repair inefficiency given the properties of these default sites (see Box 1). ChIP-Seq, chromatin immuno-precipitation followed by sequencing; DSB, DNA double strand break; PRDM9, PR domain-containing protein 9.

In recent years, several studies have evaluated in vivo the number of potential PRDM9-binding sites targeted by the DSB machinery by detecting DNA ends to which DMC1 is bound (DMC1 ChIP). Estimates count about 15,000 distinct DSB hotspots in the mouse and 30,000 in the human genome, respectively [59, 62, 63, 65, 72, 73]. However, the cytological count of DSB repair events (DMC1 foci) reveals only about 200–400 DSBs per cell [74]. This implies that only a fraction of hotspots is used in any given cell, with a different usage from cell to cell. Cytologically, thousands of PRDM9 foci, which potentially represent PRDM9 bound to its target sites, are detected in mouse early prophase spermatocytes [55, 59, 73], suggesting that PRDM9 is in large excess compared to 200–400 DSBs actually formed. Consistent with this, the total level of bound PRDM9 can vary by several fold, without altering significantly the total number of *Prdm9*-dependent DSB hotspots detected by DMC1 ChIP. This indicates a buffering effect of the DSB machinery downstream of site designation by PRDM9 [59, 61, 62, 72, 75–77]. However, several observations suggest that the number of sites bound by PRDM9 could become limiting in some specific genetic contexts. Indeed, in the C57BL/6 mouse strain (B6, carrying the *Prdm9*^*Dom2*^ allele) homologous chromosome synapsis, which depends on interactions mediated by DSB repair, displays subtle defects in *Prmd9*^*+/*−^ compared with wild type *Prmd9*^*+/+*^. This suggests that in the B6 genetic background PRDM9 could be only in slight excess compared to the required amount (discussed in section 3.3) such that reducing *Prdm9* gene dosage by half could affect DSB formation and/or repair [75]. The limiting activity of PRDM9 in B6 could be due to the extensive erosion of its hotspots that leads to a population of sites with average low affinity in the B6 genome (Fig 4) [59–61, 72, 75]. PRDM9-binding activity could be limiting also in some intersubspecific hybrids (Fig 5), whose fertility phenotypes are sensitive to *Prmd9* gene dosage [78]. As mentioned above, the number of DSBs is likely controlled by the DSB machinery downstream of PRDM9 function and several regulations have indeed been shown to coordinate DSB activity with respect to the preceding DNA replication phase and to downstream events such as homologous engagement [79]. This is illustrated by the competition for DSB formation that takes place in mice expressing together two PRDM9 variants with distinct DNA sequence specificities [60, 62, 65, 72, 75, 77]. In this situation, a semidominance effect results in DSBs forming predominantly at the sites bound by one PRDM9 variant at the expense of the other. To account for this competition and for properties of overexpressed PRDM9 in cultured somatic cells, it has been suggested that PRDM9 could bind DNA as a multimer [75, 80]. However, this property does not appear to hold in vivo in mouse spermatocytes, where differences between PRDM9 variants in their average affinity for their genomic binding sites explain readily the observed semidominance for DSB formation [77].

### PRDM9-dependent epigenetic modifications

The in vitro histone methyltransferase activity of PRDM9 is corroborated in vivo by the local enrichment of H3K4me3 and H3K36me3 at PRDM9-defined recombination hotspots [45, 56, 59, 61–63, 65, 73, 81]. The enrichment of H3K4me3 and H3K36me3 at recombination hotspots is independent of SPO11 [56, 59] but not detected in transgenic mice that express the Y357F mutation in the PRDM9 SET domain, indicating that the deposition of these marks directly depends on the catalytic activity of PRDM9 [77]. The distribution of PRDM9-dependent H3K4me3 and H3K36me3 is highly correlated, and the strongest enrichment is observed at nucleosomes that are immediately adjacent to the PRDM9-binding site [45, 82]. This enrichment is asymmetric at most hotspots with a higher level on nucleosomes either 5′ or 3′ to the PRDM9 motif [64, 82]. Furthermore, it has been proposed that PRDM9 could participate in establishing a nucleosome-depleted region at the center of hotspots. Indeed, micrococcal nuclease (MNase) profiles in spermatocytes show the generation of a stable, PRDM9-dependent nucleosome density pattern at hotspots [61]. Together, these observations reinforce the idea that PRDM9 locally promotes a unique modification of the chromatin state.

Therefore, H3K4me3 and H3K36me3 could be hallmarks for the recruitment of the DSB machinery. However, differently from H3K4me3, H3K36me3 is depleted at promoters [83], which become preferential targets of the DSB machinery in the absence of PRDM9 (Box 1). Since DSB repair is partially compromised in the absence of PRDM9 in mice, H3K36me3 could play a role in repair rather than DSB formation. It has been suggested that in somatic mammalian cells, H3K36me3 is involved in promoting the homologous recombination pathway of DSB repair, possibly by favoring the recruitment of factors involved in end resection [84–87]. Alternatively, H3K36me3 may be an incidental product of PRDM9 activity, and defects in DSB repair in *Prdm9*^−*/*−^ mice may be due to another problem. The potential role of H3K4me3 is discussed in the following section.

### Interactions between PRDM9-binding sites and the DSB machinery

**Fig 6:**
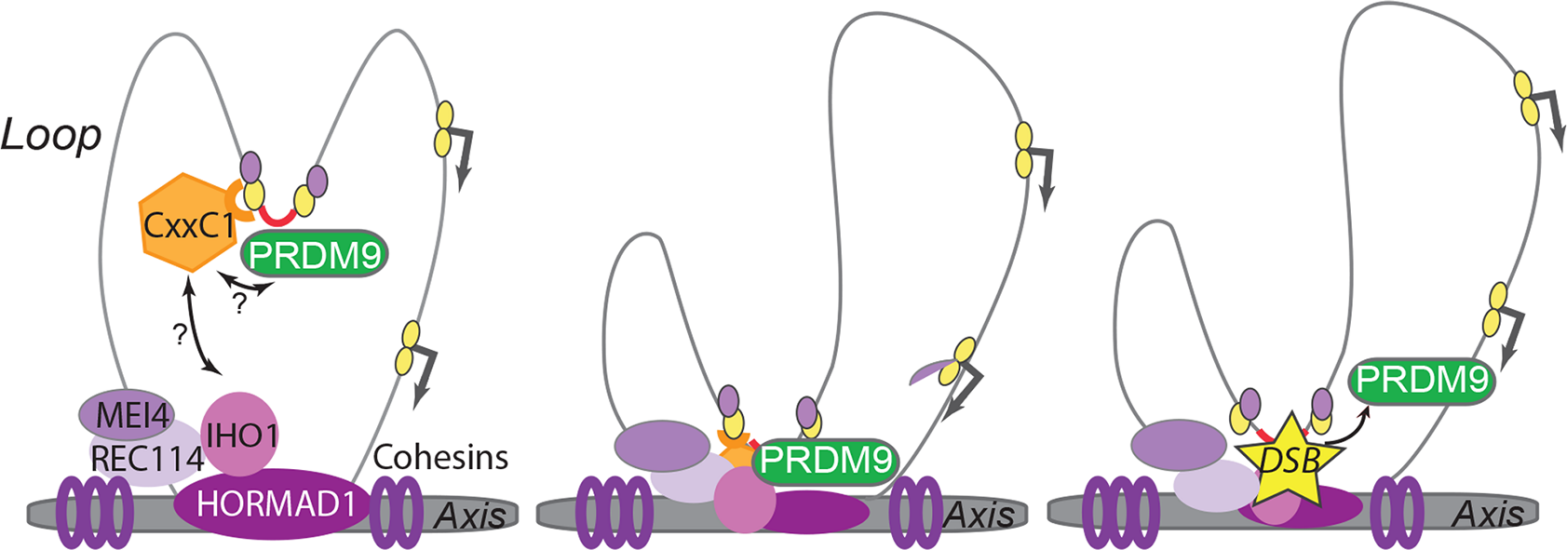
Model of PRDM9 binding dynamics in mouse spermatocytes. In early meiotic prophase, when PRDM9 is expressed, chromosomes are organized in a characteristic loop axis structure, shaped by cohesins and other proteins. Some essential components of meiotic DSB formation, such as HORMAD1, MEI4, REC114, and IHO1, are located on the axis where they form discrete foci. PRDM9 binds via its zinc finger domain to DNA motifs (red) that define meiotic recombination hotspots and that are likely to be located in chromatin loops (only one sister chromatid is represented). PRDM9 modifies the surrounding nucleosomes by catalysing H3K4me3 (yellow) and H3K36me3 (purple) deposition (left panel). Then, a reader or adaptor protein mediates PRDM9 interaction with the DNA DSB formation machinery located on the axis. This reader could be CXXC1, a H3K4me3 reader that interacts with both PRDM9 and IHO1 (central panel). Therefore, PRDM9 can also indirectly interact with DNA sequences near or on the axis. SPO11 (the protein carrying the catalytic activity for meiotic DSB formation) may be recruited at PRDM9-binding sites before or after the loop axis interaction. Upon DSB formation, PRDM9 could be displaced and could interact with other sites, such as transcription start sites, possibly through interaction with other unknown factors (right panel). DSB, DNA double strand break; H3K4me3, histone H3 lysine4 trimethyl.

In addition to histone post-translational modifications, the regulation of DSB formation is also linked to the three-dimensional organization of meiotic chromosomes. At the onset of meiotic prophase, at the time of PRDM9 binding and before DSB formation, meiotic chromosomes adopt specific higher order structural features in which sister chromatids organize into a series of loops that are anchored at their bases along a structure called the axial element (Fig 6) [88, 89]. Staining for PRDM9 in early meiocytes suggests that most PRDM9 localizes to chromatin loops [55, 59]. However, several essential components of the mammalian DSB machinery, such as MEI4, REC114, and IHO1, localize to chromosome axes where they form distinct foci [90–92]. Although the molecular activities of these proteins are not known, they are thought to activate directly the catalytic activity of the SPO11–TOPOVIBL complex for DSB formation [93, 94]. Several elegant studies in *Saccharomyces cerevisiae* involving mapping of DSB sites, recombination proteins, and axis proteins have led to a model whereby DSB sites are tethered to the axis for DSB formation [95, 96]. It has been proposed that tethering is mediated by yeast Spp1 that can interact both with H3K4me3 at hotspots through its plant homeodomain (PHD) finger domain and Mer2 (orthologue of IHO1 [97]), a member of the DSB machinery localized on the axis [98, 99]. The mouse orthologue of Spp1 is CXXC finger protein 1 (CXXC1, also called CFP1), a member of the complex of proteins associated with SET1 (COMPASS) that also contains a PHD finger domain. Two independent studies have recently identified this protein as a PRDM9-interacting partner in yeast two hybrid (Y2H) screens [49, 55]. Interestingly, CXXC1 interacts also with IHO1 in a Y2H assay [49]. This suggests that the interaction of CXXC1 with PRDM9, H3K4me3, and IHO1 could be needed to guide PRDM9-bound sites to the proximity of the DSB machinery (Fig 6). Several lines of evidence support this view; some axis proteins interact with PRDM9 [55], and potential axis sites, including CCCTC-binding factor (CTCF) sites, interact with PRDM9 in vivo [59]. Interestingly, PRDM9 and SET1 interact with overlapping domains of CXXC1, suggesting a potential competition. Consequently, the interaction of CXXC1 with PRDM9 may titrate CXXC1 away from transcription start sites, preventing DSB formation at these sites [49]. Before or upon DSB formation, PRDM9 is predicted to be displaced from its binding sites. In agreement with this, recent high-resolution DSB maps in mouse spermatocytes showed that DSBs often occur within PRDM9-binding motifs [64].

## Evolution of PRDM9 and its binding sites

### Hotspot erosion

**Fig 7:**
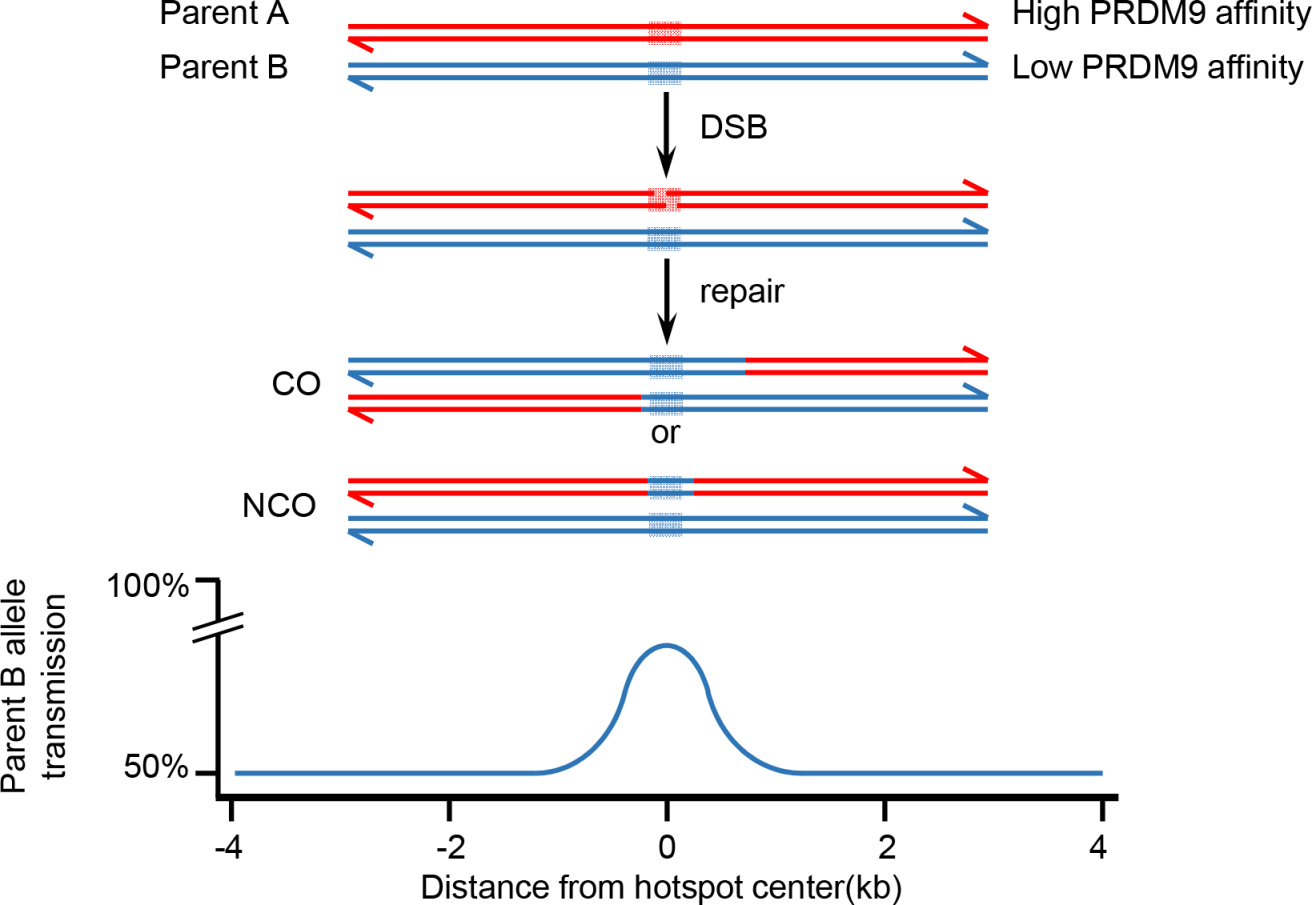
Evolution of PRDM9-binding sites. Mechanism of hotspot erosion by DSB-induced biased gene conversion. The repair by homologous recombination of a DSB leads to the replacement by gene conversion of the interval around the DSB site by the sequence from the unbroken chromatid. If two alleles with different affinity for PRDM9 are present in a population, this will result in overtransmission of the low-affinity site (blue shaded area) and erosion of the high affinity site (red shaded area). Therefore, the transmission frequency of alleles within and around PRDM9-binding sites can be higher than the 50% expected by Mendelian inheritance in the absence of bias. This effect is limited by the size of the gene conversion tracts (a few hundred bp) (lower panel). DSB, DNA double strand break; PRDM9, PR domain-containing protein 9.

Through DSB-induced biased gene conversion (dBGC), recombination occurring recurrently at hotspots can potentially lead to the disruption of hotspot activity over time (Figs 4 and 7), a phenomenon that has been referred to as the hotspot paradox [100–102]. This paradox applies when determinants for hotspot activity are located within gene conversion tracts, which is the case for PRDM9-dependent hotspots. Indeed, PRDM9-binding motifs are frequently included in gene conversion tracts [64, 68, 81, 103–106], and DSB frequency can be affected by polymorphisms in binding sites that alter their affinity for PRDM9 [16, 23, 25, 81, 104].

In agreement with these observations, the erosion of PRDM9-binding motifs at recombination hotspots has been documented by comparing the sequences of hotspots in closely related species that do not share PRDM9 alleles. These analyses revealed a consistent trend toward the loss of PRDM9-binding motifs recognized by PRDM9 alleles present in the species under study [4, 107]. Additional evidence for hotspot erosion has been brought by the experimental mapping of DSB hotspots (or of PRDM9-dependent H3K4me3 sites) in first generation (F1) hybrids from crosses of two mouse subspecies. In these hybrids, DSB hotspots specified by one *Prdm9* allele are less numerous and weaker on the chromosomes of the same origin than on the chromosomes derived from the other subspecies. This reflects the erosion of the PRDM9-binding sites specified by a given allele in the genome having coevolved with this allele [60, 72, 73].

Erosion is correlated with the strength of hotspots and does not lead to their brutal extinction but rather to the homogenization of hotspot strength by progressively weakening the activity of the strongest hotspots [105, 107]. An analysis of hotspot evolution in the human lineage suggests that the strongest hotspots are expected to disappear within 100,000 generations (3 million years) [107].

### PRDM9 diversification by mutation

PRDM9 evolution is complex. The structure of the protein is well conserved among vertebrates, indicating a function subject to selection. Interestingly, the zinc finger array of complete PRDM9 orthologues, containing all identified domains (KRAB-related, SSXRD, PR/SET, and zinc finger array) and thought to be involved in specifying recombination sites, show evidence of rapid evolution. Nevertheless, PRDM9 has suffered a total or partial (some domains) loss in several lineages independently [42, 70, 71]. Contrary to complete orthologues, partial PRDM9 orthologues show no evidence of rapid evolution of their zinc fingers, although their level of conservation suggests that they might have a function other than the specification of recombination sites [71]. The PRDM9 orthologues identified outside of vertebrates have not been carefully examined, but a putative orthologue with a zinc finger array shows evidence of rapid evolution in the cnidaria *Nematostella vectensis* [70].

The evolution of the PRDM9 zinc finger domain is shaped by the minisatellite structure of the region of the *Prdm9* gene encoding it; it is made of a single exon containing an array of 84 bp almost perfect tandem repeats that each encode a single zinc finger [7]. The diversity of PRDM9 zinc fingers presents three facets that can be related to its evolution.

First, unequal crossovers between repeats are expected to be frequent and to lead to changes in repeat numbers and can be at the origin of the observed intraspecific diversity in the number of zinc fingers (Fig 2A). The rate of changes in this number has been measured in human sperm and shown to be higher than 10^−5^ per generation [108]. This is consistent with the observed *Prdm9* allelic diversity in human populations. It suggests that *Prdm9* alleles have a shorter average lifespan than the bulk of recombination hotspots they specify.

Second, germ line recombination by unequal crossover and gene conversion within the array can lead to homogenization of zinc finger sequences within the array. This explains why zinc fingers at different positions within the array are more similar between them than individual zinc fingers from two species, even if they are at the same position within the array, a phenomenon referred to as concerted evolution [42, 109]. Thus, PRDM9 zinc finger arrays frequently contain more than one copy of the same zinc finger (examples in Fig 2A).

Third, germ line mutations can generate new zinc finger alleles. Those are expected to appear at a frequency dictated by the mutation rate, which is much lower than the rearrangement rate [110]. Those mutations have the ability to expand in the population by genetic drift and/or selection. A striking characteristic of PRDM9 zinc finger evolution is that most polymorphisms within the sequences encoding PRDM9 zinc fingers result in changes in the amino acids that are involved in DNA interaction and therefore modify the motif recognized by PRDM9. The predominance of polymorphisms that affect DNA recognition shows that the DNA-binding properties of PRDM9 alleles are under positive selection [70, 109].

### Selection

As mentioned above, intra- and interspecies polymorphisms within PRDM9 zinc fingers are characterized by a high proportion of polymorphisms that affect DNA recognition, indicating that the DNA-binding properties of PRDM9 have been under positive selection [42, 70, 109]. The erosion of recombination hotspots has been proposed as the selective pressure favoring the rise of new PRDM9 alleles that specify novel sets of hotspots [4]. The interplay between fast-eroding recombination hotspots and the rise of novel *Prdm9* alleles has been proposed to be accounted for by the model of the “Red Queen” dynamics. This model describes dynamics similar to an arms race, in which the erosion of hotspots favors the rise of novel *Prdm9* alleles that generate new sets of hotspots, which are subject to erosion in turn, with the ultimate consequence of renewing continuously and rapidly the recombination landscape [111]. The “Red Queen” dynamics of recombination hotspot evolution have been analysed by theoretical modeling [112].

As even minor changes in the order of zinc fingers in the array may lead to major reassignments of DSB hotspots [27, 41, 60, 72], PRDM9 rapid evolution could be sufficient for resetting the genomic pool of hotspots before their erosion reaches a critical threshold. While recurring rearrangements within the array are expected to reduce over time the diversity of zinc fingers within the array, appearance of a single new zinc finger by mutations has the potential of giving rise to a large number of novel PRDM9 alleles upon rearrangements within the array. The selection pressure on residues involved in DNA binding might therefore occur on a longer timescale than that of rearrangement-generated hotspot renewal in response to their erosion.

Interestingly, the function of many KRAB–zinc finger proteins consists in repressing parasitic elements, such as retrotransposons [15, 52, 113]. In many cases, the evolution of classical KRAB–zinc finger protein families involves gene duplications. One copy of the gene remains under purifying selection in order to retain the original function of repressing one specific element. Another copy is submitted to rapid adaptive evolution through frequent changes in the number of zinc fingers and positive selection on residues involved in DNA sequence recognition [14]. PRDM9 zinc finger evolution follows a similar framework but without gene duplication. Indeed, PRDM9 evolution is enhanced in response to the erosion of its targets (the recombination hotspots). In addition, some specific PRDM9 alleles may be subject to purifying selection. Indeed, specific problems may occur for instance when PRDM9 triggers DSB formation in repeated DNA sequences that could increase the risk of nonallelic genome rearrangements [114, 115]. The impact of meiotic DSBs specified by some *Prdm9* alleles and occurring within various repeat DNA families in human [22, 27, 116] and mouse [82] remains to be evaluated.

DSBs form in *Prdm9*^−*/*−^ mouse meiocytes in seemingly normal numbers but at “default sites” that share properties with DSB sites used in species lacking a functional *Prdm9* gene, such as Canidae and birds (Box 1). This raises the question of why *Prdm9* is essential for completing meiotic prophase and fertility in mice. It has been proposed that the occurrence of DSBs at too many genomic functional elements involved in controlling gene expression may be deleterious [62]. An alternative hypothesis suggests that efficient repair and homologous chromosome synapsis might require the formation of DSBs at sites with the simultaneous binding of PRDM9 on both homologues (one suffering the DSB, the other not). This hypothesis was proposed to account for the phenotypes of sterile hybrid mice in which a substantial fraction of DSBs are formed at sites heterozygous for PRDM9 binding and show synapsis and apparent partial DSB repair defects [73]. Restoration of homozygosity over large genomic regions indeed restored *in cis* synapsis defects in such hybrids [117] (Fig 5). More subtle effects due to such heterozygosity could be more widespread and have an impact on reproductive isolation in wild populations. For instance, the failure of a limited number of chromosomes to pair and synapse can be sufficient to compromise meiotic progression in males. Therefore, these findings suggest that a decrease in PRDM9-dependent function, from DNA binding to DSB formation and potentially DSB repair, could lead to a decrease of fitness by lowering fertility.

The controlled localization of recombination events and their dynamics is an outstanding property with respect to the consequences on genome stability, genome rearrangements, and association of alleles. In humans, *PRDM9* is the first hit in genome-wide association studies on recombination distribution, but there is no established evidence for a link to fertility. However, an association has been detected between human *Prdm9* zinc finger polymorphism and reduced recombination on Chromosome 21 observed in cases of Chromosome 21 nondisjunction [118]. One may speculate that different PRDM9 zinc finger arrays could have distinct numbers of binding sites on a given chromosome, which could impact the rate of recombination. Several coding polymorphisms in the *PRDM9* gene are significantly more frequent in men with azoospermia caused by meiotic arrest [119, 120], but their causal implication has not been shown. The recent identification of a healthy and fertile mother who carries two loss-of-function *PRDM9* alleles argues against an essential role in human fertility [121].

PRDM9, by defining the location of recombination hotspots, might also play an important role in other aspects of human health linked to genome instability. In several diseases caused by nonallelic homologous recombination (NAHR), such as Charcot-Marie-Tooth disease, X-linked ichthyosis, Hunter and Potocki–Lupski/Smith–Magenis syndromes, disease-associated breakpoints that contain a PRDM9 hotspot at their center have been identified [22, 27, 65, 122, 123]. Moreover, some rare *PRDM9* variants have been linked to the development of acute lymphoblastic leukaemia in children [124, 125], but *PRDM9*’s role in this disease is unclear.

## Concluding remarks

PRDM9 orthologs have been detected in vertebrates and in several nonvertebrates, suggesting that the PRDM9 family is ancestral to bilaterians [10, 70]. However, many species, including some vertebrates, have lost PRDM9 and have a distinct control of the localization of meiotic DSBs, which appears to rely on features that promote access to DNA of the meiotic DSB machinery, including chromatin modifications and accessibility. PRDM9 substitutes and suppresses this pathway by three fundamental properties: the ability to bind to multiple sites in the genome, the generation of a proper chromatin environment with the modification of histones (H3K4me3 and H3K36me3), and protein interactions that, in a process still poorly understood, lead to activation of DSB activity. The PRDM9-dependent and -independent pathways are thus two distinct molecular strategies to initiate the meiotic recombination program, which is by itself largely conserved among sexually reproducing eukaryotes. However, these two pathways have two major distinct consequences on meiotic recombination, which are its distribution along the genome and the evolution of this distribution. In the absence of PRDM9, sites of meiotic recombination are mostly located in proximity to genes or regulatory regions, whereas in the presence of PRDM9, recombination sites are distributed within genic and intergenic regions, a pattern that also depends on the DNA binding specificity of PRDM9 zinc finger array. In the long term, in the absence of PRDM9, DSB sites are relatively stable in the genome, whereas in the presence of PRDM9, as a consequence of hotspot erosion and selection of novel PRDM9 variants, DSB localization evolves extremely rapidly. The question of what the advantage is, if any, of one pathway versus the other remains open. Given the negative impact of PRDM9 on its own activity, one may assume that this pathway has or had some selective advantage. This advantage may be transient, as several vertebrate species have lost PRDM9. One potential long-term consequence of the distribution of recombination could be its impact on the linkage between regulatory regions and genes. Regulatory regions in the genome are most often accessible regions of the chromatin, therefore the absence of PRDM9 meiotic recombination will tend to disrupt linkage between regulatory elements and genes. Interestingly, it has been recently proposed that a specific selective process may act onto regulatory elements, which is called the Enhancer runaway [126]. This process is due to selection for optimal expression levels and is most efficient when regulatory regions are in *cis* and linked to the genes they regulate [127]. It thus appears that controlling the pattern of recombination with PRDM9 may increase the efficiency of the Enhancer runway, and this could be favorable in some contexts. This hypothesis remains to be tested, and other scenarios could certainly be envisioned to understand the long-term consequences of the distribution of meiotic recombination and the potential contribution of PRDM9 in this process.
